# Skin Protection Behaviors among Young Male Latino Day Laborers: An Exploratory Study Using a Social Cognitive Approach

**DOI:** 10.1155/2016/1479637

**Published:** 2016-01-04

**Authors:** Javier F. Boyas, Vinayak K. Nahar, Robert T. Brodell

**Affiliations:** ^1^School of Social Work, University of Alabama, Box 870314, Tuscaloosa, AL 35487, USA; ^2^Department of Health, Exercise Science & Recreation Management, University of Mississippi, 215 Turner Center, P.O. Box 1848, University, MS 38677, USA; ^3^Department of Dermatology, University of Mississippi Medical Center, 2500 N. State Street, Jackson, MS 39216, USA; ^4^Department of Pathology, University of Mississippi Medical Center, 2500 N. State Street, Jackson, MS 39216, USA; ^5^Department of Dermatology, University of Rochester School of Medicine and Dentistry, Rochester, NY 14642, USA

## Abstract

Latino Day Laborers (LDLs) are employed in occupations where multiple work hazards exist. One such hazard is the overexposure to solar ultraviolet radiation for continuous periods of time. Regular sun exposure can put individuals at increased risk of developing skin cancers, especially without adequate protection. The purpose of this cross-sectional exploratory study was to use a social cognitive framework to assess skin protective behaviors among LDLs. A community-based nonrandom and purposive sample of LDLs was recruited in two states: Mississippi and Illinois. The study sample consisted of 137 male participants, of which the majority were of Mexican ancestry (72%). The average age was 35.40 (SD = 9.89) years. Results demonstrated that a substantial number of LDLs do not adequately practice sun protection behaviors on a regular basis. The skin cancer knowledge scores were very modest. The most frequently indicated barriers towards sun protection were “inconvenient,” “forget to use,” and “not being able to reapply sunscreen.” Overall, LDLs had moderate confidence in their abilities to adopt successful sun protection strategies. This study underscores the need for intervention programs aimed at LDLs to reduce extended time in the sun and increase use of sun protective measures when working outdoors.

## 1. Introduction

The workplace is an overlooked determining factor of health and health disparities [1]. Work determines a person's income, health insurance benefits, and psychosocial functioning, all of which influence individual health [1–3]. However, it also relates to how much an individual is exposed to varying types of occupational hazards. It is well established that Latino Day Laborers (LDLs) are often employed in occupations that are highly hazardous, highly stressful, and low paying, are exposed to poor working conditions, and lack health insurance coverage [2–5]. Research on the risks associated with occupational exposures for LDLs continues to grow. Research suggests that LDLs are usually hired for jobs that involve a number of workplace threats, such as working with unsafe mechanized tools and equipment, environments with high noise levels, exposure to dangerous chemicals, work in risky heights, lack of personal protective equipment, and little to no safety oversight [2, 6]. However, the hazard of regular exposure to solar ultraviolet radiation (UVR) is one area that has remained completely unexplored. Continuous contact with UVR is commonplace for LDLs given that they are most frequently employed in job sectors such as landscaping, gardening, roofing, painting, and construction [5, 7]. These work activities commonly take place outdoors, which expose LDLs to considerable sun contact.

Overexposure to UVR for continuous periods of time, without proper protection, during peak periods of intense sun, can put individuals at risk of developing melanoma or other nonmelanoma forms of skin cancers [8]. Skin cancer is one of the top 10 new cancer diagnoses among adults in the USA [9, 10], and incidence rates in the USA have continued to soar in the past 35 years [11]. Among Latinos, incidence rates of melanoma are on the rise and they have risen at an annual rate of 2.9%, which is comparable to rates among non-Hispanic whites [12]. This is problematic given that only one in fourteen Latinos reports ever having a physician skin examination [13]; only 3.2% of Latinos have been told how to perform a skin self-examination [14]; and Latinos, relative to non-Hispanic whites, were less likely to wear sun protective clothing or sunscreen with a protection factor of 15 or higher [14]. These findings may partially explain why Latinos are often diagnosed at an earlier age and are diagnosed at advanced stages of melanoma and often have poorer cancer survival rates than non-Hispanic whites [9, 15]. Thus, research is needed that can assist this high risk and vulnerable population in the prevention of skin cancer.

Our research is important given that LDLs are typically employed in outdoor jobs, such as construction, landscaping, and framing, where UVR exposure is continuous. LDLs who are outdoor workers often experience regular exposure to solar UVR for extended periods of time, which can place this population at an increased risk of developing skin cancer [16–18]. Despite this recognition, to the best of our knowledge, no published studies have investigated the extent to which LDLs engage in skin protective behaviors. Thus, this is the first exploratory study that seeks to establish the sun protective perceptions and practices of LDLs. Our study utilized social cognitive theory (SCT) to help us understand cognitive and social factors that contribute to skin protective behaviors of LDLs [19]. The data collected as part of this study was based on social cognitive theory applied to health behavior [19, 20]. Social cognitive theory suggests that social and physical environments influence behavior. Moreover, there are incessant reciprocal exchanges among people and their environments and behaviors [19]. The purpose of this study was to use a social cognitive framework to identify psychosocial forms of self-efficacy in relation to employing sun protective strategies, intention to protect oneself from the sun, perceived workplace support, and perceived barriers and benefits associated with skin protective behaviors.

## 2. Methods

### 2.1. Sample and Procedures

The current study utilized cross-sectional data from the 2014 Mississippi Survey on Health Practices. To participate in this study, possible participants had to self-identify as either Hispanic or Latino, be at least 18 years of age, actively seek informal and short-term contingent employment, have no cognitive limitations, be not institutionalized, and reside in Mississippi or Illinois. In all, we surveyed a total of 138 participants: 77 of the participants were from Mississippi, whereas 61 were from Illinois. One of the participants was a female day laborer. However, she was deleted from the analysis.

A community-based nonrandom and purposive sample of Latino participants was recruited in two states: Mississippi and Illinois. In Mississippi, the sample was recruited from multiple cities, including Hernando, Holly Springs, Oxford, and Southaven. The recruitment strategy varied in each state. In Mississippi, we relied on multiple strategies. The participants were recruited through advertisements (English and Spanish) and referrals from collaborating Latino/Hispanic serving social and health care providers. Recruitment flyers were posted at sites that day laborers frequently visit, such as Mexican* taquerias*, Laundromats, Catholic churches, and construction sites. In addition, face-to-face recruitment methods were also employed at various construction sites. A snowball technique was also used. We asked all participants if they could recommend someone else who was appropriate for the study. This strategy was beneficial given that day laborers are considered a hard-to-track population. This is especially true in some of the smaller southern towns where this population does not have a long history nor can they congregate on street corners or building supply store parking lots like they would in metropolitan areas. In Illinois, the sample was recruited from three common street corners where day laborers gather in the northwest side of Chicago. In Illinois, we approached LDLs seeking employment during the morning hours.

Data were collected from July 2014 to November 2014 from various urban, rural, and semirural communities in Mississippi and Illinois. This time frame allowed us to examine the summer sun protective behaviors in order to avoid a possible recall bias. In Mississippi, there are more extended periods of sunshine and warm temperatures that carry over well into the fall season. As such, data collection continued until November. However, in Illinois, data collection stopped in early October. That is because, in the Midwest, the number of sunny and warm days becomes less as the fall approaches. Informed consent authorizing participation in this study was provided to participants along with a self-report questionnaire. The questionnaire was available in English and Spanish. An option of an oral reading of the survey was offered to all participants. This measure was taken to ensure that potential respondents with literacy concerns did not have to disclose that they were illiterate and were not discouraged from participating in the survey. Study participants voluntarily completed a roughly 55-minute self-administered questionnaire developed primarily from established and validated instruments. Participants were given the opportunity to fill out the survey before or after work and received a $20.00 research honorarium. Members of the research team, which included the principal investigator and three bilingual/bicultural research assistants, administered data collection. Prior to the administration of the survey, all research assistants were trained for two hours on culturally sensitive data collection procedures. Institutional Review Board permission was obtained in order to ensure minimal risk to study participants.

### 2.2. Measures

A survey instrument was developed utilizing established scales and measures from prior research [21–29]. Some of these instruments were available in Spanish [26]. If a Spanish version was a not available, the research team, which consisted of two Mexican Americans, one Peruvian, and one Venezuelan, translated the instruments. All translations were first carried out independently. Then, the group discussed how to best structure the items, with the intention of making the language suitable to a population that is characterized by low levels of educational attainment.

A total of nine items were used to identify sun protection behaviors (e.g., wide-brimmed hat, long-sleeved shirt, shirt with collar, limiting midday sun, sunglasses, sunscreen, gloves, and covering head and face) [26]. These items were measured using a five-point scale (1 = “never”; 5 = “always”). For example, “During the summer months at work, how often do you wear sunscreen with sun protection factor (SPF) of 15 or higher when you are in the sun for more than 15 minutes?”; additionally, information on sunscreen use behavior (e.g., thorough application, SPF values, and reapplication) was assessed by three questions [26].

To assess perceived benefits of sun protection strategies, participants were asked to rate their level of agreement (1 = “strongly agree”; 5 = “strongly disagree”) with three statements [21, 23]. The questions, for example, focused on “decrease the risk of skin cancer” and “decrease skin aging.”

Twelve Likert-rated (1 = “strongly disagree”; 5 = “strongly agree”) statements were used to assess perceived barriers among respondents for not engaging in sun protection behaviors [21, 23]. Three example statements are “I want to get a suntan,” “sun protective clothing is too hot to wear,” and “sunscreen is greasy.”

Self-efficacy (i.e., the degree of confidence that an individual has in his/her ability to conduct a particular behavior) scale consisted of seven items that asked participants about their confidence level to use recommended sun protection measures (e.g., seeking shade, wide-brimmed hat, long-sleeved shirt, long pants, sunglasses, and sunscreen) [28–31]. Response categories ranged from 1 (“not at all confident”) to 10 (“highly confident and certainly can do”).

LDLs were asked about their sources of information (i.e., television, radio, newspaper, health care workers, family, friends, coworkers, and employer and supervisor) regarding protecting themselves from too much sun [21]. For each of the 9 items, responses were measured on a three-point scale (1 = “yes”; 0 = “no”).

Knowledge regarding skin cancer (nonmelanoma skin cancer and melanoma) and risk factors was assessed by 24-item scale developed by Cottrell and colleagues (2005) (1 = “correct”; 0 = “incorrect”) [25]. For example, “The most common form of skin cancer is - Basal cell carcinoma or Squamous cell carcinoma or Melanoma?” The total possible score for knowledge ranged from 0 to 24, with a higher score indicating higher skin cancer related knowledge.

Intentions to engage in sun protection practices (e.g., applying sunscreen, wearing protective clothing, long-sleeved shirt, seeking shade, and using sunglasses) were measured by five questions, using a five-point scale (1 = “strongly agree”; 5 = “strongly disagree”). Participants were asked if they would intend to use sun protection at work every time they go in the sun for more than 15 minutes during the summer months.

The authors developed the two workplace support items. We asked LDLs to state how much they think their supervisor(s) engage(s) in sun protective behaviors. They were also asked to assess how much they think their coworkers engage in sun protective behaviors. Both items were 5-point Likert-type coded as follows: 1 = never; 5 = always.

Skin screening was assessed by three questions [22]. The first question assessed if they ever had their skin checked for changes, which could be skin cancer (1 = “yes”; 0 = “no”). The second question assessed who checked their skin (1 = “I did,” 2 = “skin doctor,” 3 = “my partner did,” 4 = “general practitioner,” 5 = “a friend did,” and 6 = “another family member did”). The third question assessed when they had their most recent skin exam (1 = “Within last year,” 2 = “1 to 3 years ago,” 3 = “Over 3 years ago,” and 4 = “I do not know”).

### 2.3. Analytic Strategy

Univariate statistics (i.e., frequencies and percentages) were used to describe all the variables collected in this study. Bivariate analyses were used to determine if statistical differences existed between data collected in Illinois and Mississippi. Additional bivariate analyses were conducted to determine if significant differences exist by legal status and education level in relation to major study constructs. The analyses were all conducted using Statistical Package for Social Sciences (SPSS) version 21 (Chicago, IL). Significance level was set at 0.05* a priori*.

## 3. Results

The sample description is presented in Table 1. The men in our sample were mainly of Mexican ancestry (72%). The second largest group was from Central America (18%). The average age was 35.40 (SD = 9.89) years. In terms of legal status, the largest group was undocumented (35%). Our sample was not highly educated; 44% reported less than an 8th grade education. The majority of the sample who were foreign born reported receiving their education in their country of origin (88%). Those participants who were born in another country have been in the USA an average of 11.15 (SD = 9.48) years. The majority of participants were married (*n* = 69). The majority lived with roommates (*n* = 53). Of those that reported having roommates, the average number of roommates was 3.86. Sixty-nine percent reported earning less than $20,000 a year in 2014. In terms of work types, the majority worked in multiple areas, such as construction, painting, roofing, and lawn mowing. However, the largest group reported working in the area of construction (42%). All of the LDLs worked for private employers. The majority of the respondents were outdoor workers who spent 4.66 (minimum 1; maximum 6) hours outside in the sun between 10 a.m. and 4 p.m. Only 7.3% of LDLs reported ever having their skin examined for changes, which could be skin cancer.

Data were examined to determine if statistical differences existed between the data collected in Mississippi and Illinois; however, no significant differences were found in any of the sun protective perception or behavior variables. Two areas where they did differ were in the areas of legal status and acculturation levels. A higher number of LDLs reported being undocumented in Mississippi compared to Illinois (*x* ^2^ = 13.084, *p* ≤ 0.05). We also found that acculturation levels of LDLs were significantly higher in Illinois (M = 6.65; SD = 3.13), compared to Mississippi (M = 5.65; SD = 2.71).

In terms of sun protective behaviors, the most frequently reported method was use of wearing something over their head, such as a hat, cap, or visor (see Table 2). The LDLs reported “always” or “often” wearing something on their head 26% of the time. The largest portion though, 41%, reported wearing something on their head only some of the time. The next two most reported sun protective practices were wearing sunglasses and wearing hats with a surrounding 2.5-inch brim. Twelve percent of respondents reported “always” or “often” wearing sunglasses, while 8% reported “always” or “often” wearing a hat with a surrounding brim of at least 2.5 inches. Some of the information on lack of practicing sun protective behaviors was concerning. For example, 59% of LDLs reported never using sunscreen, while 76% reported never wearing any protective gear over their face, such as a handkerchief or filter mask. More than half of the sample also reported never wearing a hat with a surrounding brim of at least 2.5 inches, gloves, a long-sleeved shirt, or a shirt with a collar.

In terms of protecting oneself from UVR by way of sunscreen with SPF 15, the majority of the sample did not report wide use. In all, only 6% reported wearing sunscreen “always” or “often.” In terms of where they apply it, 35% reported applying sunscreen to their face. Another 28% applied sunscreen to their neck, 22% applied it to their ears, 27% applied it to their upper arms, 34% applied it to their lower arms, and 24% applied it to their hands. The highest area to be left unprotected by sunscreen was the legs; 88% reported not applying sunscreen to that part of their body. On the day of data collection, only 17% were wearing sunscreen. When asked how many times a day they applied sunscreen when they are at work, their average was low (M = 0.61; SD = 1.03).

Levels of self-efficacy in relation to using different practices to protect themselves from the sun varied among LDLs (see Table 3). Collectively, our indicators suggest that LDLs had moderate confidence in their abilities to use various strategies to protect themselves. The highest level of confidence was professed for wearing long pants (M = 6.89; SD = 2.83), wearing sunglasses (M = 6.13; SD = 3.32), and wearing work gloves (M = 5.60; SD = 3.49). The two areas that LDLs were least confident in using were using sunscreen (M = 4.75; SD = 2.69) and seeking shade (M = 4.98; SD = 2.51).

In terms of perceived barriers, LDLs reported having multiple barriers to protecting themselves from the sun between 10 a.m. and 4 p.m. (see Table 4). The top three barriers were as follows: not always being convenient to protect oneself from the sun (M = 4.17; SD = 0.74), often forgetting to protect oneself from the sun (M = 4.16; SD = 0.90), and not being able to reapply sunscreen (M = 4.12; SD = 0.64). Other barriers included sunscreen sweating off of them, sun protective clothing being too hot to wear, sun protective measures were too time consuming, and being concerned about sun exposure. LDLs did not believe that sunscreen was too feminine (M = 2.85; SD = 1.00) or that sunscreen had a bad smell (M = 2.93; SD = 0.87). LDLs were neutral on sun protective measures being expensive, sunscreen being greasy, and sunscreen attracting dirt.

Turning to benefits of protecting themselves while out in the sun for more than 15 minutes between 10 a.m. and 4 p.m., LDLs were asked about doing so to decrease skin aging, decrease sunburn, and decrease the risk of developing skin cancer. In terms of aging, 64% either “strongly agreed” or “agreed” that this was a perceived benefit of protecting themselves from the sun. Sixty-one percent of LDLs either “strongly agreed” or “agreed” that a perceived benefit of protecting themselves from the sun was to decrease the likelihood of sunburn. Last, 73% of LDLs reported protecting themselves from the sun to decrease the risk of developing skin cancer.

LDLs were also surveyed about their future intent to engage in a series of sun protective behaviors. The strongest intention reported was to seek shade during the peak hours of the day (M = 3.55; SD = 1.24). They were least likely to report future intentions to engage in using sunscreen with SPF 15 or better (M = 2.80; SD = 1.22). They were more neutral about intending to wear protective clothing, such as hats, wear long-sleeved shirts, and wear sunglasses.

LDLs were also surveyed about their perception of whether their supervisors and coworkers engage in sun protective behaviors. Our results suggest that LDLs perceived that neither their supervisors (M = 2.50; SD = 1.02) nor coworkers (M = 2.66; SD = 1.13) often engage in sun protective behaviors.

With regard to receiving information about sun protection, television (88.2%) and radio (77.1%) were indicated most frequently by participants, followed by family (62%), friends (47.1%), newspapers (46.6%), coworkers (39.3%), health care workers (37.4%), and supervision (34.1%).

We sought to also establish how much LDLs knew about skin cancer and whether they could identify risk factors associated with melanoma. The knowledge scores were very modest. The overwhelming number of the participants reported not knowing answers to the questions. In terms of identifying risks associated with melanoma, we asked if they could identify them from 8 different risk factors. Collectively, LDLs were able to identify a modest number (M = 2.60; SD = 1.55). Given this lack of knowledge, we examined whether achieved education levels could partially explain this result. However, we found that education was only significantly associated with self-efficacy (*t* = 2.178; *p* ≤ 0.05), but not SPBs (*t* = −1.020; *p* ≥ 0.05); barriers (*t* = 1.170; *p* ≥ 0.05); or being able to identify skin cancer risk factors (*t* = −0.402; *p* ≥ 0.05). LDLs with some college level training beyond a high school diploma were able to express higher levels of self-efficacy (M = 44.72; SD = 17.24) in relation to using sun protective strategies, compared to LDLs with a high school diploma or less (M = 37.90; SD = 17.48).

Given that Mississippi included more persons who were not legal, we examined whether legal status shaped SPBs, barriers, self-efficacy, and being able to identify skin cancer risk factors. We collapsed the categories so that LDLs who reported being legal (citizen by birth, naturalized citizen, and permanent legal resident) were in one category and LDLs with a work permit and nonimmigrant visa and noncitizen nor permanent legal resident were grouped in another. Significant differences were found in the areas of SPBs (*t* = 5.709; *p* ≤ 0.001) and being able to identify skin cancer risk factors (*t* = 5.252; *p* ≤ 0.001). LDLs who held a legal status reported using more SPBs (M = 19.36; SD = 5.78), compared to LDLs who were not legal (M = 14.60; SD = 3.85). Legal LDLs were able to properly identify more skin cancer risk factors (M = 3.40; SD = 1.67), compared to LDLs who were not legal (M = 2.17; SD = 1.31). Legal status did not have a significant effect on reporting barriers or self-efficacy.

## 4. Discussion

We used a social cognitive approach to identify the practices and beliefs of sun protection behaviors among LDLs. Our results suggest that sun protection practices are necessary given the length of time LDLs are exposed to UVR. On average, LDLs spend 4.66 hours outdoors during peak periods of sun intensity. Further complicating matters, only 7.3% of the respondents had ever had their skin examined, despite continuous UVR contact. Thus, LDLs are exposed to dangerous levels of UVR, without any attention given to protection or having their skin examined. This finding is consistent with the broader literature that suggests that very few Latinos have ever had a physician skin exam [13]. These two findings call for health promotion professionals to assist LDLs in protecting themselves out in the field, by educating them about the harms and risks associated with continuous exposure to UVR during peak periods of intense sun. They could also assist in educating them about the benefits of having skin exams. Although encouraging LDLs to have physician skin exams is preferred, it is not likely given that so many of them do not have access to health care resources because they are uninsured. Thus, at the very least, LDLs should be trained on how to perform self-skin examinations. This might be one strategy that could help; however, it has to be a health message that has to be conveyed to this population continuously. In one study, Latinos in one southern state reported that they were not told by their physicians to get self-skin examinations, which is why they did not perform them [12]. Thus, getting reminders from health professionals may help LDLs adhere to having skin examinations on a continual basis.

In terms of sun protective behaviors, LDLs reported concerning levels of unsafe sun practices. They reported a low frequency of wearing protective clothing during summer. LDLs also indicated that they did not do much to limit the sun exposure at midday. Furthermore, many of the LDLs reported not using sunscreen with SPF 15. Our findings suggest that they were not currently applying sunscreen and it was unlikely that this practice would change in the future. A large portion reported never applying sunscreen and many also reported that their future intent to engage in a series of sun protective behaviors did not include applying sunscreen. The reported levels of sunscreen use and wearing a long sleeve shirt were much lower among LDLs compared to other reports of sun protective behaviors among US Latinos [32, 33], and other studies focused on outdoor workers [34]. LDLs reported higher levels of using hats compared to broader Latina/o samples [32]. This finding can be partially explained by the low levels of acculturation of our sample, which could point to a cultural contrast in norms among LDLs. It has been maintained that sunscreen is a US cultural norm [32] that may not be reflective of norms in other cultures or countries. Varying degrees of sun protective behaviors have been linked to differences in acculturation levels among Latinos. For example, Andreeva and colleagues (2009) found that Latinos with lower levels of acculturation were significantly less likely to use sunscreen compared to those who were more acculturated [32]. In that study, lower levels of acculturation had a negative effect on sun protective behaviors, such as applying sunscreen. However, other research suggests that lower levels of acculturation can have a protective effect as well. Coups and colleagues (2013) found that less acculturated Latinos were more likely to wear sun protective clothing and seek shade but were less likely to report sunbathing and indoor tanning [33]. These contradictory findings suggest that the relationship between acculturated Latinos and sun protective behaviors is complex.

Poor sun-safe practices reported by LDLs may be attributed to a lack of knowledge. In this study, LDLs demonstrated that they lacked knowledge about skin cancer and they were not able to identify risk factors associated with melanoma, which is consistent with findings of the broader Latino population in terms of skin cancer knowledge [35]. The knowledge scores that were obtained in this study were not usable data because the overwhelming majority of respondents stated that they did not know the answer or provided an incorrect answer. Our measure on identifying risk factors further corroborated this knowledge gap. This finding establishes that education and awareness about skin cancer and risk factors are necessary among LDLs. Despite this recognition, prevention interventions that target LDLs and the broader Latina/o population remain inadequate [35]. More interventions are needed that can target the Latina/o population as a whole but also that address the unique workplace risks faced by LDLs. Given that the majority of LDLs receive sun protection information from television and radio, it would be beneficial for skin cancer agencies to collaborate with media channels in order to develop effective sun safety programs.

The barriers reported by LDLs should be taken into account as interventions are being developed. Our findings suggest that LDLs may not have the option of seeking shade, stopping work to reapply sunscreen, or thinking about how to better protect oneself from the sun. These are barriers that should be addressed through interventions that target these areas. For example, what can be done to protect oneself when time and flexibility are an issue and there are not many ways to stop work to think about seeking shade? These barriers should likely be addressed structurally through public policies. It may be that the US Occupational and Safety Health Administration (OSHA) has to take a more proactive role in considering the harmful effects of UVR as a legitimate occupational risk hazard. This position is not very pronounced in their information on personal protective equipment [36]. Their informational document does not address specifically the type of gear and/or equipment needed to properly protect oneself from UVR.

The LDLs reported varying levels of self-efficacy in regard to sun protective practices when in the sun for more than 15 minutes during peak times. Most reported moderate levels of confidence in wearing sun protective clothing. However, they were less inclined to make compromises towards wearing sunscreen and seeking shade. Not wearing sunscreen was consistently reported across various measures used in this study. However, it is not known whether the reported levels of self-efficacy towards using sunscreen are low due to an outright unwillingness to apply it or because it is part of the descriptive norms in which they work. It has been maintained that descriptive norms have a powerful influence on behavior in ways that may lead individuals to adopt risky health behaviors [37]. In regard to lacking self-efficacy in seeking shade, it may be that LDLs lack self-efficacy because they are mainly outside workers and they have very little latitude in terms of stopping work merely to take a break from the sun. This may cause them to be dismissed and then face the challenge of securing another job. LDLs may be willing to work through continuous exposure to UVR and overlook this risk simply because of the need to keep their job. The economic pressures faced by LDLs can make them averse to leaving a job, even if it is hazardous [38]. These findings warrant additional research to look into identifying what strategies could be used to enhance the self-efficacy and motivation of LDLs to regularly apply sunscreen and seek shade whenever possible during work hours. It is recommended that interventions be designed using Motivational Interviewing (MI) as an intervention approach to help LDLs strengthen their level of self-efficacy in relation to sun protective behaviors in order to reduce the number of unsafe sun practices, such as not applying sunscreen and not seeking shade. MI-based prevention interventions have been successful in enhancing self-efficacy and motivating the readiness of participants to practice safer sex and ensuring positive behavior change [39].

The workplace environment appears to be another type of barrier. Supervisors and coworkers were not identified as a frequent source of information about protecting from too much sun. Moreover, LDLs reported not having high beliefs that their supervisors and coworkers engage in sun protective behaviors. This can influence them to potentially espouse the same blasé attitude towards sun-safe practices and behaviors. Other research has established that descriptive norms predict sun protection behaviors [40]. Supervisors and coworkers may socially reject the use of sun protective strategies. This behavior can be demonstrated indirectly by not using or discussing sun-safe practices. This may be especially true in the environment where LDLs work, where supervisors' and coworkers' norms regarding sun protective behaviors serve as group-level referent informational influences [40]. Health promotion professionals should consider whether conducting sun safety educational training sessions at worksites could be a viable path in modifying workplace descriptive and injunctive norms in support of greater use of sun protection practices. Mobile training, like clinics, may be a viable approach to promote better sun protective practices among LDLs. This training should not just target the workers but the supervisors as well. Supervisors can play an integral part in shaping the attitudes and behaviors workers exhibit in the workplace. They themselves can model the behaviors that promote more adherence to using sunscreen practices. In general, the Latino culture strongly supports respect for authority figures, such as supervisors [41]. This cultural trait can be used positively. The respect for authority figures may facilitate recommendations made by supervisors to increase use of sun-safe practices among LDLs [41].

## 5. Limitations

This research is subject to some limitations. Due to the small sample size and nonrandom sampling design, the generalizability of the findings to all LDLs may be limited. We believe participation in this research was impacted by recent anti-immigrant public policies in neighboring states, Alabama and Georgia. Latino immigrants experience a great deal of stress and fear in relation to being “discovered” and then deported [42]. Even LDLs who are legal may have opted out of participating because they may have feared being harassed or exploited. It is likely that the sociopolitical climate could have exacerbated their fear and discouraged LDLs from participating. Further, the majority of the participants in this study were males recruited from only two states; therefore, sample may not be entirely representative of all LDLs. Future studies should involve diverse LDL samples collected from larger geographic areas. Another limitation of our study is that findings are reliant on self-report responses, which might have introduced recall bias in the study. However, previous studies have validated self-report of sun protection using observational methods [43, 44]. Moreover, we tried to increase the sun protection recall accuracy by collecting data during summer months or a little after the end of summer months. Lastly, the majority of our sample reported low levels of acculturation. This issue could have influenced the overall results. Despite these limitations, this study provides firsthand information critically required to develop specific intervention programs to improve adoption of skin cancer prevention behaviors among this population group.

## 6. Conclusions

In summary, the findings of this study suggested that a substantial number of LDLs, in particular those who are not legal, do not adequately practice sun protection behaviors on a regular basis. Our results underscore the need for intervention programs aimed at LDLs to reduce extended time in the sun and increase use of sun protective measures when working outdoors. This is important given that LDLs continue to work in areas where the exposure to levels of UVR remains high. Moreover, since overall knowledge of skin cancer among this occupational group was considerably low, LDLs should be specifically educated on their vulnerability of future skin cancer risk and importance of comprehensive preventive strategies. Hence, future interventions should incorporate components to effectively minimize LDLs' barriers towards sun protection and improve their self-efficacy in wearing sunscreen and protective clothing, especially because SPBs are malleable behaviors. Doing so may provide a path for reducing the risk of developing skin cancer amongst such a susceptible group.

## Figures and Tables

**Table 1:** Sociodemographic characteristics of the Latino Day Laborers.

	*n *(%)
*Ethnic background*	
Colombian	1 (0.7%)
Cuban	1 (0.7%)
Ecuadorian	6 (4.5%)
Guatemalan	11 (8.2%)
Honduran	9 (6.7%)
Mexican	97 (72.4%)
Nicaraguan	1 (0.7%)
Peruvian	2 (1.5%)
Puerto Rican	3 (2.2%)
Salvadoran	3 (2.2%)
*Racial background*	
White/Caucasian	34 (24.8%)
African American	1 (0.7%)
Indigenous	50 (36.5%)
More than one race	48 (35%)
*Legal status*	
United States citizen	16 (11.9%)
Naturalized citizen	12 (8.9%)
Permanent legal resident	19 (14.1%)
Work permit	24 (17.8%)
Nonimmigrant visa	14 (10.4%)
Noncitizen and not permanent legal resident	50 (37%)
*Level*	
Less than elementary school	61 (44.5%)
Completed elementary school but not high school	27 (19.7%)
High school diploma	2 (1.5%)
Associate degree	41 (29.9%)
Bachelor's degree	5 (3.6%)
Graduate or professional degree	1 (7%)
*Skin type*	
Always burn, never tans	29 (21.5%)
Usually burn, tans with difficulty	5 (3.7%)
Sometimes mild burn, gradually tans to a light brown	24 (17.8%)
Rarely burn, tan with ease to a moderate brown	45 (33.3%)
Very rarely burns, tans very easily	17 (12.6%)
Never burns, tans very easily, deeply pigmented	15 (11.1%)
*Educated in native country*	
Yes	115 (83.9%)
No	22 (16.1%)
*Marital status*	
Single	45 (33.3%)
Married	69 (51.1%)
Separated	4 (3%)
Divorced	3 (2.2%)
Widowed	1 (7%)
Living with partner	13 (9.6%)
*Living arrangements*	
Roommates	53 (39.8%)
Spouse	15 (11.3%)
Spouse and children	23 (17.3%)
Relatives	21 (15.8%)
Live-in partner	11 (8.3%)
Alone	10 (7.5%)
*Type of work*	
Construction	57 (41.6%)
Roofing	4 (2.9%)
Landscaping	25 (18.2%)
Painting	15 (10.9%)
Cementing	2 (1.5%)
Lawn mowing	8 (5.8%)
Clean-up	1 (0.7%)
Welding	1 (0.7%)
Multiple	24 (17.5%)
*Health insurance coverage*	
Yes	21 (16.3%)
No	108 (83.7%)
*Household income (yearly)*	
Less than $20,000	95 (69.3%)
$21,000 to $30,000	41 (29.9%)
$31,000 to $40,000	1 (0.7%)

	Mean (SD)

Age (years)	35.40 (9.89)
Lived in USA (years)	11.15 (9.48)

**Table 2:** Sun protection behaviors of Latino Day Laborers.

	Never	Sometimes	About half the time	Often	Always
	*n *(%)	*n *(%)	*n *(%)	*n *(%)	*n *(%)
Wear something on your head (any type of hat, cap, or visor)	8 (5.8%)	56 (40.9%)	37 (27%)	24 (17.5%)	12 (8%)
Wear hat with a surrounding brim of at least 2.5 inches	71 (51.8%)	40 (29.2%)	15 (10.9%)	6 (4.4%)	5 (3.6%)
Wear a long-sleeved shirt	76 (55.5%)	45 (32.8%)	14 (10.2%)	1 (0.7%)	1 (0.7%)
Wear shirt with a collar	70 (51.1%)	52 (38%)	10 (7.3%)	4 (2.9%)	1 (0.7%)
Limit the time you are exposed to the sun at midday	49 (35.8%)	59 (43.1%)	26 (19%)	3 (2.2%)	0 (0%)
Wear sunscreen with SPF of 15 or higher	81 (59.1%)	33 (24.1%)	15 (10.9%)	6 (4.4%)	2 (1.5%)
Wear sunglasses	43 (31.4%)	56 (40.9%)	21 (15.3%)	11 (8.0%)	6 (4.4%)
Wear gloves	73 (53.3%)	49 (35.8%)	11 (8%)	4 (2.9%)	0 (0%)
Wear any protective gear over your face (hankie, filter mask)	104 (75.9%)	27 (19.7%)	6 (4.4%)	0 (0%)	0 (0%)

**Table 3:** Sun protection self-efficacy of the Latino Day Laborers.

	Mean (SD)
Seek shade	4.98 (2.51)
Wear a wide-brimmed hat with a surrounding brim of at least 2.5 inches	5.35 (3.22)
Wear a long-sleeved shirt	5.51 (3.34)
Wear long pants	6.89 (2.83)
Wear sunglasses	6.13 (3.32)
Wear work gloves	5.60 (3.49)
Wear sunscreen with a sun protection factor (SPF) of 15 or higher	4.75 (2.69)

**Table 4:** Sun protection barriers of the Latino Day Laborers.

	Mean (SD)
Not concerned about sun exposure	3.74 (0.93)
Sun protection clothing is too hot to wear	3.99 (0.86)
Not always convenient to protect myself from the sun	4.17 (0.74)
Often forget to protect myself from the sun	4.16 (0.90)
Sun protection measures are expensive	3.60 (0.93)
Use of sun protection measures is time consuming	3.85 (0.60)
Use of sunscreen is too feminine	2.85 (1.00)
I don't like the smell of sunscreen	2.93 (0.87)
Sunscreen is greasy	3.45 (1.09)
Sunscreen attracts dirt	3.46 (1.06)
Sunscreen sweats off of me	4.04 (0.77)
I can't reapply sunscreen	4.12 (0.64)
